# Generative Artificial Intelligence Literacy: Scale Development and Its Effect on Job Performance

**DOI:** 10.3390/bs15060811

**Published:** 2025-06-13

**Authors:** Xin Liu, Longxin Zhang, Xiaochong Wei

**Affiliations:** School of Public Administration and Policy, Renmin University of China, Beijing 100872, China; lxin@ruc.edu.cn (X.L.); longxin@ruc.edu.cn (L.Z.)

**Keywords:** Generative Artificial Intelligence literacy, Creative Self-Efficacy, job performance, scale development, Ability–Motivation–Opportunity theory

## Abstract

With the rapid development of generative artificial intelligence, its application in the workplace has shown significant innovative potential and practical value. However, the existing literature lacks a systematic and widely applicable definition and measurement framework for Generative AI Literacy. Based on the existing literature and following a rigorous scale development process, this study constructs a Generative AI Literacy measurement framework that covers five core dimensions, basic technical competence, prompt optimization, content evaluation, innovative application, and ethical and compliance awareness, and validates its reliability and validity. Furthermore, based on the Ability–Motivation–Opportunity (AMO) theory, this study explores the mechanism through which Generative AI Literacy influences employee job performance and examines the mediating role of Creative Self-Efficacy. The results show that Generative AI Literacy has a significant positive impact on job performance (*β* = 0.680, *p* < 0.001), with Creative Self-Efficacy playing a partial mediating role (indirect effect = 0.537). The developed five-dimensional framework demonstrates strong psychometric properties and provides empirical evidence for AI literacy’s role in enhancing workplace performance through Creative Self-Efficacy mechanisms. This study provides an effective measurement tool for research on the application of Generative AI Literacy in workplace settings and offers practical insights for organizations to optimize performance and promote the responsible use of AI.

## 1. Introduction

Generative Artificial Intelligence (GAI) is rapidly reshaping social structures, economic systems, and organizational management models, offering powerful momentum for global digital transformation. As a key branch of artificial intelligence, GAI can autonomously generate multimodal content such as text, images, audio, and video, demonstrating broad application prospects across domains including education, healthcare, and management (Ng et al., 2024; Pan et al., 2024). This technological breakthrough not only enhances productivity and decision-making quality but also fosters novel innovation paradigms, accelerating intelligent transformation across industries. Unlike traditional AI technologies that primarily focus on analysis and prediction, GAI enables autonomous content creation and comprehension through deep learning and natural language processing, marking a revolutionary shift in productivity tools and creativity empowerment. It is important to recognize that AI technologies do not operate as autonomous systems; rather, they require human intelligence for guidance, critical evaluation, and ethical oversight. The human role as a mediator in AI applications makes Generative AI Literacy an essential capability in the digital age (Kwon, 2024). While GAI offers significant opportunities for efficiency improvements and innovation in organizational contexts, it also presents challenges including data privacy concerns, algorithmic bias risks, employee skill displacement, and ethical dilemmas. Understanding this dual nature is crucial for responsible AI deployment and organizational success. These challenges underscore the importance of cultivating Generative Artificial Intelligence Literacy (GAIL) as a critical factor for both individuals and organizations to effectively utilize GAI (Bozkurt, 2024a; Lee & Park, 2024).

GAIL refers to the comprehensive set of capabilities required by individuals and organizations in the use of generative AI technologies, encompassing technical operation, creative application, critical evaluation, ethical awareness, and collaborative communication (Ng et al., 2024; Lee & Park, 2024). The theoretical foundation of GAIL draws from several established scholarly traditions, including digital literacy theory (Ng et al., 2024), social cognitive theory (Bandura, 2001), technology acceptance model (Davis, 1989), and critical thinking theory (Ennis, 1993). The concept of GAIL stems from the intersection of digital literacy theory and the technology acceptance model, but it has evolved into a distinct theoretical construct due to the unique interactive mechanisms, creative potential, and ethical challenges posed by generative AI. GAIL not only emphasizes the ability to use technology but also highlights users’ capacity for collaborative innovation with AI systems and their critical and ethical engagement with AI-generated content. This expands beyond the traditional boundaries of digital literacy and technology acceptance models.

Despite growing scholarly attention, current research on GAIL faces three systematic limitations that hinder both theoretical advancement and practical application. First, the theoretical foundation of GAIL remains fragmented and lacks systematic conceptual integration. The selection of five core dimensions in this study—basic technical ability, prompt optimization, content evaluation, innovative application, and ethical compliance—is grounded in established theoretical rationales. Basic technical ability stems from the foundational principles of digital literacy theory (Ng et al., 2024); prompt optimization embodies the unique characteristics of human–AI collaborative interaction (Oppenlaender et al., 2023); content evaluation reflects the application of critical thinking frameworks in AI contexts (Tang et al., 2023); innovative application corresponds to creativity and innovation demands in contemporary knowledge work (Bilgram & Laarmann, 2023); and ethical compliance embodies responsible AI usage principles and regulatory compliance requirements (Bozkurt, 2024a). For example, the ABCE framework proposed by Ng et al. (2024)—emphasizing Affective, Behavioral, Cognitive, and Ethical dimensions—was primarily developed for educational contexts and may not be fully applicable in complex workplace environments. Inconsistencies in the definition of GAIL’s conceptual dimensions have led to theoretical fragmentation, hindering both theoretical advancement and empirical accumulation in the field. Some studies reduce GAIL to technical skills, overlooking the role of critical thinking, while others overemphasize ethics at the expense of practical application. This conceptual inconsistency poses a considerable barrier to the development of robust theoretical models.

Second, existing measurement tools for GAIL lack generalizability and broad applicability. Many current instruments are tailored to specific user groups such as teachers, students, or technical managers. For instance, the ChatGPT Literacy Scale developed by Lee and Park (2024) focuses on highly educated users, limiting its applicability across diverse organizational and industrial contexts (Wang et al., 2023; Lintner, 2024). Moreover, these tools often lack validation across cultures and have limited predictive validity in dynamic AI application scenarios.

Third, although prior studies have suggested that GAIL positively influences individuals’ trust in technology, creativity, and learning capabilities (Baabdullah, 2024), the mechanisms through which GAIL contributes to employee performance in organizational contexts remain underexplored. Recent research has further indicated a lack of cross-cultural validation and limited adaptability of current tools to dynamic scenarios (Lintner, 2024; Wang et al., 2023), underscoring the need to develop scalable, cross-role, and context-sensitive measurement systems for future research.

To address these systematic limitations, this study adopts the Ability–Motivation–Opportunity (AMO) framework to investigate how GAIL influences job performance through the interplay of individual ability (A), motivational factors (M), and organizational opportunities (O) (Blumberg & Pringle, 1982; Appelbaum, 2000). Rather than employing multiple competing theoretical frameworks, this study establishes a focused dual-core theoretical architecture: the AMO framework serves as the primary explanatory framework for understanding how GAIL influences job performance, while social cognitive theory (Bandura, 2001) provides the psychological foundation for understanding the mediating mechanisms, particularly Creative Self-Efficacy. This approach avoids theoretical fragmentation while maintaining analytical depth. The AMO theory offers several advantages as a core theoretical lens for this study. First, unlike linear skill–performance models, the AMO framework provides a multidimensional and systemic explanation for how complex technical capabilities translate into job performance. Second, its emphasis on the interactive effects among ability, motivation, and opportunity aligns well with the multifaceted nature of GAI application, where effective use depends not only on technical competence but also on user intention and organizational support. Third, the AMO framework has been extensively applied in digital transformation and innovation management research and demonstrates strong explanatory power in emerging domains such as generative AI. Widely employed in strategic human resource management and high-performance work systems (HPWS), the AMO model has proven to be a valuable tool for understanding employee performance (Jiang et al., 2012).

In GAI contexts, the effectiveness of GAIL depends not only on individual technical proficiency but also on users’ motivation and the availability of organizational support. Specifically, individuals with high GAIL are more likely to exhibit trust in AI and creative engagement in the workplace. However, whether such advantages can be translated into improved performance hinges on motivational drives and the availability of appropriate organizational resources. Thus, the AMO theory offers a comprehensive framework to examine how GAIL operates within organizational settings and to uncover the potential mediating role of psychological variables such as Creative Self-Efficacy (CSE).

The selection of Creative Self-Efficacy as a key mediating variable is theoretically grounded in the integration of social cognitive theory (Bandura, 2001) and the AMO framework. social cognitive theory posits that Self-Efficacy is a crucial psychological mechanism that translates capability into action. The motivational dimension of the AMO model aligns closely with this mechanism. In the context of GAI, the translation of technical literacy into performance outcomes is not solely determined by technical skills, but also by individuals’ confidence in their creative use of such technology. As a bridge between GAIL and job performance, Creative Self-Efficacy helps explain the mechanism of this relationship and illuminates the pathway through which technological empowerment fosters psychological empowerment and ultimately enhances performance.

Accordingly, this study aims to address the identified theoretical and practical gaps through three specific objectives: (1) developing a comprehensive measurement framework for workplace Generative AI Literacy that captures the multidimensional nature of AI-human collaboration; (2) conducting exploratory factor analysis and confirmatory factor analysis with private sector employees to validate the reliability and validity of the developed scale; and (3) performing empirical analysis with public sector employees to examine the mechanism through which GAIL influences job performance and the mediating role of Creative Self-Efficacy. First, drawing on the established dual-core theoretical framework, this study proposes an integrative model that systematically delineates the structure and developmental pathways of GAIL. Based on common workplace demands, this study identifies core dimensions of GAIL and, leveraging both the existing literature and natural language processing (NLP) technologies, develops a more objective, precise, and generalizable measurement tool. Second, two rounds of data collection are conducted with private sector employees to perform exploratory factor analysis (EFA) and confirmatory factor analysis (CFA) to assess the reliability and validity of the developed scale. Third, using the AMO framework, this study conducts empirical analysis with public sector employees to examine both the direct effect of GAIL on job performance and its indirect effect via Creative Self-Efficacy. This cross-sectoral research design enhances the robustness and generalizability of the findings, demonstrating the scale’s applicability across diverse organizational environments. By integrating theoretical modeling with empirical analysis, this study offers both theoretical insights and practical guidance for the responsible use and capability development of generative AI in organizational contexts, while laying the groundwork for future investigations into GAIL’s relationship with other organizational variables.

## 2. Development of the Generative AI Literacy Scale for the Workplace

### 2.1. Literature Review

Generative Artificial Intelligence (GAI) is built upon deep learning and large-scale data training techniques. It enables the creation of multimodal content—including text, images, audio, and video—with a high degree of autonomy and adaptability across diverse scenarios (Cao et al., 2023). From a technological evolution perspective, GAI represents a paradigm shift from “analytical intelligence” to “creative intelligence,” transforming technical architectures and redefining human–AI collaboration. Unlike traditional AI that emphasizes classification and prediction, GAI is oriented toward content creation and innovation. For instance, ChatGPT generates high-quality natural language text, while DALL·E produces diverse images based on user prompts, offering unprecedented creative support (Cao et al., 2023). Notably, the role of GAI in the workplace has evolved from that of a simple auxiliary tool to a catalyst for innovation, reshaping workflows, decision-making models, and modes of creative expression. However, widespread GAI adoption has raised issues including privacy concerns, algorithmic bias, and ethical misuse, imposing higher standards for responsible use (Tang et al., 2023).

Contemporary organizational research emphasizes the pressing need for systematic AI literacy development in workplace contexts. Studies indicate that inadequate employee AI competencies represent a significant barrier to digital transformation initiatives, with skill gaps identified across sectors (Abou Karroum & Elshaiekh, 2023). Cross-industry research demonstrates that AI literacy gaps affect organizational innovation capacity and competitive advantage, highlighting the need for comprehensive workplace-oriented AI literacy frameworks (Borines et al., 2024).

In response to these challenges, researchers have increasingly recognized that a lack of user competence in handling GAI technologies is a major bottleneck limiting its effective implementation. Inadequate prompt engineering skills may result in low-quality or irrelevant outputs, while insufficient critical evaluation capabilities hinder users’ ability to assess the logical coherence and reliability of generated content (Dang et al., 2022; Lin, 2023). Additionally, limited awareness of ethical and compliance issues can further exacerbate the risks of misuse (Tang et al., 2023). These issues collectively underscore the importance of cultivating Generative AI Literacy (GAIL). GAIL is proposed as a theoretical construct aimed at assessing users’ multifaceted capabilities in employing GAI, thereby supporting both technological effectiveness and social responsibility.

GAIL is theoretically rooted in established scholarly traditions that provide a foundation for scale development. First, it draws from the traditions of information literacy and digital literacy, emphasizing the ability to identify, evaluate, and utilize information effectively within digital environments (Machin-Mastromatteo, 2021). Second, it incorporates insights from the technology acceptance model (TAM) and the diffusion of innovation theory, which explore users’ psychological mechanisms and behavioral tendencies regarding technology adoption (Y. Zhou, 2008). Third, GAIL integrates key perspectives from critical thinking theory, which emphasizes rational evaluation and reflective judgment of AI-generated content (Gonsalves, 2024). Finally, it incorporates elements from ethical decision-making theory, foregrounding the importance of ethical considerations and value-based judgment in technology use (Mertz et al., 2023). This multi-theoretical integration provides a solid foundation for the GAIL framework and reflects its multidimensional nature.

To understand current GAIL measurement landscapes and identify theoretical gaps, this study conducted a comprehensive comparative analysis of existing frameworks. This comparative analysis not only reveals the diversity of existing approaches but, more importantly, exposes some key limitations that partially demonstrate the necessity of developing workplace-specific frameworks. Existing studies have made preliminary efforts to conceptualize and measure GAIL, but several important distinctions indicate the necessity and value of developing alternative approaches.

Specifically, Ng et al. (2024) proposed the ABCE framework—Affective, Behavioral, Cognitive, and Ethical—outlining four core dimensions of GAIL through a 32-item validated questionnaire targeting secondary school students. Their approach, grounded in educational psychology frameworks, emphasizes the balance between emotional engagement and technical competence in educational settings. However, this framework’s focus on student learning outcomes rather than workplace performance competencies somewhat limits its applicability to organizational contexts where collaborative innovation and professional standards are important.

Meanwhile, Lee and Park (2024), focusing specifically on ChatGPT users, developed a multidimensional 25-item scale that includes technical proficiency, critical evaluation, communication proficiency, creative application, and ethical competence. Their methodology employed the Delphi method for expert consensus and focus group validation, offering valuable insights into user experience with specific AI platforms. However, the platform-specific nature of their framework may limit generalizability across diverse GAI technologies, and their emphasis on individual user experience may not adequately address the collaborative and organizational performance requirements that characterize workplace AI implementation.

Among existing AI literacy frameworks, Nong et al. (2024) merit particular attention due to their broad dimensional scope and focus on the general public. The authors proposed a comprehensive seven-dimensional model that includes application ability, cognitive ability, morality, critical thinking, Self-Efficacy, perceived ease of use, and perceived usefulness. Their framework is designed for the general population and emphasizes perceptual measures grounded in technology acceptance theory, validated through large-scale surveys and structural equation modeling. However, compared with the workplace-oriented framework proposed in this study, several limitations emerge. First, in terms of conceptual validity, potential conceptual overlap between dimensions such as “application ability” and “Self-Efficacy” may undermine discriminant validity. Second, regarding the measurement focus, the emphasis on perceptual constructs (e.g., perceived ease of use and usefulness) reflects users’ attitudes rather than actual behavioral competencies required in professional settings, thereby limiting the framework’s predictive validity for workplace performance. Third, in terms of contextual applicability, a general population focus may not sufficiently account for the collaborative dynamics, regulatory compliance, and performance standards that are critical in workplace AI adoption. Finally, from the perspective of theoretical integration, while the framework draws from multiple theoretical traditions, it lacks a tightly integrated structure, which may constrain its overall explanatory power.

Additionally, Zhao et al. (2022) developed a four-dimensional framework encompassing “Knowing and Understanding AI,” “Applying AI,” “Evaluating AI Application,” and “AI Ethics,” specifically targeting primary and secondary school teachers. Their research, based on educational technology frameworks and validated through structural equation modeling analysis with 1013 survey participants, found that “Applying AI” had significant positive effects on the other three dimensions. While their teacher-specific needs assessment and expert content validation provide valuable insights, the occupation-specific design and educational context constraints somewhat limit cross-sector applicability and may not adequately address the broader organizational requirements that characterize contemporary workplace AI implementation.

To provide a systematic comparison of approaches in this field and highlight the unique contributions of this study’s framework, Table 1 presents a comprehensive analysis of major GAIL scale development studies, detailing comparisons of their theoretical foundations, methodological approaches, target populations, and related limitations.

As shown in Table 1, the GAIL framework proposed in this study differs significantly from existing models of AI literacy in its multidimensional structure, demonstrating notable strengths in both theoretical construction and practical relevance. First, in terms of competency architecture, this study adopts a process-oriented design that conceptualizes GAIL as a dynamic and progressively developing competency spectrum. The framework moves from foundational technical understanding to advanced creative application, reflecting the iterative and evolving nature of human–AI collaboration in workplace environments. This developmental perspective stands in contrast to traditional models that often treat AI literacy as a static collection of discrete skills, offering a more theoretically grounded and future-oriented approach. Second, regarding the integration of technical and ethical dimensions, this framework departs from prior instruments that typically emphasize either technical proficiency or educational application. Instead, it seeks to achieve a balanced synthesis of technical capability and ethical responsibility across all dimensions. This dual emphasis aligns with the real-world demands of professional environments, where the ability to competently and responsibly engage with AI technologies is increasingly essential. Finally, in terms of contextual adaptability, this study is explicitly tailored to the workplace, incorporating key organizational considerations such as collaborative innovation, regulatory compliance, and performance accountability. These elements, often overlooked in general-purpose or education-focused frameworks, enhance the contextual relevance of GAIL for contemporary organizational settings.

A deeper analysis of current research on the theory and measurement of Generative AI Literacy (GAIL) reveals three primary conceptual gaps that this study attempts to address, which to some extent demonstrates the necessity and value of the proposed framework. First, regarding conceptual boundaries, further clarification may be needed. Some existing studies may not adequately distinguish GAIL from general AI literacy, to some extent overlooking the unique interactive mechanisms and creative attributes of generative AI. This may result in some degree of conceptual overlap and ambiguity. Second, the theoretical connotations of GAIL require systematic development. Most existing frameworks may be constructed more based on practical needs rather than theoretical guidance, potentially leading to relatively unclear logical relationships and developmental pathways among dimensions. Third, measurement frameworks exhibit some imbalance. Current scales often may overemphasize either technical operation or ethical awareness, failing to adequately achieve balanced representation of technical capability and social responsibility, which may to some extent affect the comprehensiveness and validity of measurement efforts. These gaps collectively underscore the importance of constructing a rigorous conceptual and measurement framework for GAIL grounded in relatively solid theory.

Based on the above analysis, this study redefines Generative AI Literacy (GAIL) as a multidimensional conceptual framework for evaluating users’ capabilities throughout the entire process of using generative AI technologies. The choice of five core dimensions—basic technical proficiency, prompt optimization, quality evaluation, innovative practice, and ethical and compliance awareness—rather than alternative configurations is grounded in both theoretical considerations and practical demands of the workplace. This dimensional design aims to address several shortcomings found in existing frameworks. Compared with the seven-dimensional model by Nong et al. (2024), which may suffer from conceptual overlap, the five-dimensional structure in this study seeks to enhance discriminant validity. In contrast to Lee and Park’s (2024) platform-specific framework, the present model strives for cross-platform and cross-technology applicability. Furthermore, while many existing models are oriented toward educational contexts, this framework is explicitly designed to align with workplace performance requirements. This redefinition offers three key theoretical advantages. First, it conceptualizes GAIL as a multidimensional capability structure rather than a singular skill set, highlighting its holistic and systematic nature. Second, it frames GAIL as a process-oriented competence, emphasizing performance across the entire life cycle of technology use rather than static knowledge acquisition. Third, it integrates technical application with ethical responsibility, uniting the ability to use AI with the capacity to use it responsibly—thereby providing a more comprehensive and practice-oriented interpretation of digital literacy.

To systematically capture the multidimensional nature of users’ competencies in using generative AI, this study identifies five core dimensions of GAIL based on extensive literature review and established theoretical traditions. The construction of the conceptual model follows established competency development theoretical frameworks, particularly Bloom’s cognitive taxonomy’s hierarchical structure, which provides a relatively clear pathway for skill development from basic cognition to advanced creation (Voinohovska & Doncheva, 2024). Meanwhile, mature research in the digital literacy field provides important reference for constructing multidimensional competency frameworks, particularly in the integration of technical capabilities and critical thinking skills (Jordan et al., 2024). These dimensions form a unified theoretical model as a relatively integrated, hierarchical competency spectrum—ranging from foundational to advanced levels, and from technical operation to ethical judgment.

The five-dimensional structural design of the conceptual model also draws from theoretical insights about competency progression in information literacy research. Research in the information literacy field indicates that relatively effective digital competency frameworks typically include core elements such as technical foundations, information processing, critical evaluation, and innovative application (Adeleye et al., 2024). This study’s dimensional selection largely aligns with this established pattern while making adjustments and extensions for the unique characteristics of generative AI. Additionally, research in technology acceptance and usage provides important theoretical support for understanding how users progress from basic use to innovative application (Johnson et al., 2014). Human–computer interaction research also provides valuable theoretical foundations for understanding GAI’s unique interaction patterns and competency requirements, particularly in prompt design and content evaluation (Shen et al., 2025).

From the perspective of cognitive development theory, this model reflects a progression from basic skill acquisition to critical thinking and ultimately to creative application. From the lens of technology acceptance and usage, it represents a deepening trajectory from understanding and use to innovation. Ethically, it embodies awareness of responsible technology use throughout the application process. This theoretical architecture facilitates systematic understanding of the internal structure of GAIL and provides a relatively clear foundation for developing corresponding measurement tools.

Basic technical proficiency is the foundational dimension of GAIL, rooted in digital literacy theory (Ng et al., 2024) and information processing frameworks (Ebert & Louridas, 2023). It refers to users’ understanding of the basic principles and operational techniques of generative AI, providing prerequisites for the development of other capabilities. Related empirical research suggests that technical foundational capabilities may directly affect the development of subsequent advanced capabilities and the effective use of AI tools (Campbell et al., 2023). In Bloom’s taxonomy, this corresponds to the “remember” and “understand” levels, serving as the basis for constructing effective mental models.

Prompt optimization ability represents a relatively unique and important skill in GAI applications, derived from human–computer interaction theory (Oppenlaender et al., 2023) and communication studies (Liu et al., 2023). It measures users’ capacity to design and refine prompts that improve the quality and relevance of generated content. This ability may directly affect the efficiency of human–AI interaction and represents an important characteristic distinguishing generative AI from traditional AI. Prompt engineering represents a shift from “using technology” to “guiding technology” and reflects metacognitive capabilities in human–machine collaboration.

Content evaluation ability emphasizes users’ critical thinking in assessing AI-generated outputs, based on critical thinking theory (Tang et al., 2023) and information literacy frameworks (Fu et al., 2023). This skill is of significant importance for ensuring the reliability and practical utility of generated content. Related empirical research indicates that content evaluation ability may serve as an important safeguard against automation bias and cognitive delegation, potentially directly affecting the quality of AI-assisted decision making.

Innovative application ability captures users’ capacity to transform creative ideas into practical outcomes using GAI, rooted in creativity theory (Bilgram & Laarmann, 2023) and innovation diffusion frameworks (Harwood, 2023). This dimension represents the higher-order component of GAIL, corresponding to the “synthesize” and “create” levels in Bloom’s taxonomy. Organizational research suggests that innovative application ability may serve as an important mediating factor in translating technological literacy into job performance, potentially to some extent determining the organizational return on AI investment.

Ethical and compliance awareness represents an important dimension within this study’s GAIL framework. This dimension addresses users’ capabilities to identify potential risks in GAI applications and adhere to relevant legal, regulatory, and social norms that characterize professional environments, drawing from ethical decision-making theory (Tang et al., 2023) and technology ethics frameworks (Banh & Strobel, 2023). In workplace contexts, this dimension becomes particularly relevant as organizations must navigate complex regulatory requirements, data privacy concerns, intellectual property considerations, and professional standards that govern AI implementation. This dimension recognizes that responsible AI use requires dedicated competencies that are essential for comprehensive Generative AI Literacy in workplace contexts.

To further explore how GAIL influences job performance and its underlying mechanisms, this study establishes a focused dual-core theoretical architecture that attempts to avoid the theoretical fragmentation observed in previous research. This study integrates the Ability–Motivation–Opportunity (AMO) framework as the primary explanatory lens for understanding how GAIL influences workplace performance, while social cognitive theory (Bandura, 2001) provides the psychological foundation for understanding Self-Efficacy mechanisms and learning processes. This integrated approach builds upon established scholarly traditions while maintaining theoretical coherence and analytical clarity, potentially offering certain advantages over existing frameworks that often lack coherent theoretical grounding. From the perspective of the dual-core theoretical architecture, the GAIL model provides a relatively solid theoretical foundation for subsequent empirical research, systematically elucidating how individual AI competencies translate into organizational performance outcomes through psychological mechanisms.

### 2.2. Item Generation Based on Artificial Intelligence Algorithms

This study employs an AI-based approach for generating and screening measurement items, utilizing modern natural language processing (NLP) models to assist in scale development. Drawing from the pioneering work of Hernandez and Nie (2023), who demonstrated the effectiveness of transformer-based models in personality scale development, we adapted their established methodology to address the specific requirements of workplace Generative AI Literacy measurement. Compared to traditional methods that rely heavily on expert intuition and subjective experience, AI-driven approaches allow for objective and highly efficient generation of candidate items—for instance, producing item pools at the scale of millions within a short time frame. AI-generated items can be evaluated across multiple dimensions simultaneously, including linguistic structure, semantic relevance, content validity, and construct coverage, thereby offering a more systematic and reproducible validation mechanism compared to conventional expert-based approaches.

The item generation pipeline consists of four sequential stages, each leveraging distinct AI capabilities to ensure both quantity and quality of generated items. The process began by clearly defining the target construct and its primary dimensions. Based on these definitions and a set of carefully selected seed items representing each GAIL dimension, item generation was directed accordingly. Following Hernandez and Nie’s (2023) validated framework, we utilized their pre-trained generative transformer model accessed through Claude 3.5 Sonnet API. This model was selected for its demonstrated superior performance in generating contextually appropriate and linguistically sophisticated items compared to earlier models like GPT-2, which, despite being freely available, tend to exhibit relatively lower accuracy in domain-specific generation tasks.

In the semantic similarity assessment stage, we implemented the BERT-based approach established by Hernandez and Nie (2023) to estimate semantic relatedness between generated items and original seed items. BERT (Bidirectional Encoder Representations from Transformers) was selected over alternative semantic similarity methods due to its bidirectional context processing capability, which enables more nuanced understanding of item semantics compared to unidirectional models. The semantic similarity was calculated using cosine similarity between BERT embeddings, with items achieving similarity scores above 0.7 with seed items being retained for further evaluation. This threshold was established based on Hernandez and Nie’s validation studies to balance semantic relevance with item diversity.

For content validation, we employed zero-shot classification models rather than traditional manual content validity indexing. This methodological choice was motivated by several considerations demonstrated in the original framework: (1) zero-shot classifiers can process large item pools efficiently without requiring extensive labeled training data; (2) they provide consistent and reproducible content categorization across different validation sessions; (3) they can assess content coverage across multiple theoretical dimensions simultaneously; and (4) they reduce potential human bias in content evaluation while maintaining high accuracy rates. The zero-shot classifier was configured to evaluate each generated item against the five GAIL dimensions, providing probability scores for dimension membership and enabling systematic content balance assessment. It is important to note that, after the AI-based item generation and screening process, the candidate items were reviewed and refined by three domain experts. They evaluated each item for conceptual clarity, construct relevance, and linguistic appropriateness, making necessary minor adjustments to improve item quality while preserving the original semantic structure generated by the AI.

Finally, the reliability and validity of the scale were evaluated through exploratory factor analysis (EFA) and Confirmatory Factor Analysis (CFA), followed by further validation of scale performance using additional samples. This multi-stage validation approach ensures that AI-generated items not only demonstrate semantic coherence but also exhibit appropriate psychometric properties for measurement applications.

### 2.3. Exploratory Factor Analysis

To validate the construct of the Generative AI Literacy Scale, this study distributed electronic questionnaires using a snowball sampling method through social networks and resources from classmates, friends, and family. The snowball sampling was initiated through the researchers’ personal networks, beginning with initial contacts in private sector organizations and asking them to refer colleagues with similar organizational backgrounds. To ensure sample homogeneity and relevance to the workplace context, we specifically targeted employees from private enterprises based on the following organizational classification criteria: respondents were asked to identify their current workplace as (1) state-owned enterprise/state-holding enterprise, (2) private enterprise, (3) foreign-invested enterprise, (4) joint venture, (5) public institution, (6) government agency, (7) social organization/non-profit institution, or (8) other. Only responses from individuals employed in private enterprises (Option 2) were included in the analysis.

A total of 311 questionnaires were distributed with a response rate of 89.4%, yielding 278 valid responses after data screening for completeness and attention checks. No formal quotas were established for demographic characteristics, but we monitored sample composition during collection to ensure adequate representation across age and experience groups. Inclusion criteria required respondents to be current employees in private enterprises with at least basic familiarity with AI technologies, while exclusion criteria eliminated responses with incomplete data or failed attention checks. In terms of gender distribution, 48.56% were male and 51.44% were female. Regarding age, 20.14% were aged 25 or younger, 38.85% were aged 26–35, 25.54% were aged 36–45, 10.79% were aged 46–55, and 4.68% were aged 56 or older. In terms of marital status, 23.74% were unmarried and single, 20.14% were unmarried but not single, 12.23% were married without children, and 43.88% were married with children. Regarding education, 4.32% had a college degree or lower, 50.72% held a bachelor’s degree, 38.49% held a master’s degree, and 6.47% had a doctoral degree. In terms of work experience, 22.30% had 5 years or fewer, 23.74% had 6–10 years, 26.26% had 11–15 years, 14.75% had 16–20 years, and 12.95% had more than 21 years. Regarding monthly income, 10.79% earned below 5000 CNY, 39.21% earned between 5000 and 8000 CNY, 30.22% earned between 8000 and 12,000 CNY, and 19.78% earned more than 12,000 CNY.

This study conducted exploratory factor analysis (EFA) on the 311 data points using SPSS 27.0. The results indicated a Kaiser–Meyer–Olkin (KMO) value of 0.915, which exceeds the recommended threshold of 0.7. Bartlett’s Test of Sphericity yielded a chi-square value of 5244.240 (*df* = 136), which was significant at the 0.1% level, indicating that the data are suitable for factor analysis. Principal component analysis was used to extract factors, with oblique rotation, and factors with eigenvalues greater than 1 were retained. The results of the EFA showed that the five extracted factors accounted for a cumulative variance of 89.746%, surpassing the 60% threshold. These five dimensions were: basic operational skills, prompt engineering ability, innovative application ability, quality evaluation ability, and compliance and ethical awareness. As shown in Table 2, the standardized factor loadings for all measurement items ranged from 0.82 to 0.92, all exceeding the 0.5 threshold, indicating good construct validity for the items.

### 2.4. Confirmatory Factor Analysis of the Scale

To assess the fit of the factors in the Generative Artificial Intelligence Literacy Scale, this study conducted a second independent data collection using the same snowball sampling methodology and organizational targeting criteria as the EFA phase. Following the same approach, we leveraged social networks of classmates and friends to identify and recruit private enterprise employees, maintaining consistency in sample characteristics with the exploratory phase.

A total of 337 questionnaires were distributed with a response rate of 90.8%, and after screening using identical inclusion and exclusion criteria as the EFA phase, 306 valid samples were obtained for confirmatory factor analysis. The same organizational classification question was used to ensure all respondents were private enterprise employees, and attention checks were implemented to maintain data quality. In terms of gender distribution, 50.33% of the respondents were male, and 49.67% were female. Regarding age distribution, 20.26% were aged 25 or younger, 38.56% were aged 26–35, 25.49% were aged 36–45, 10.78% were aged 46–55, and 4.90% were aged 56 or older. In terms of marital status, 19.28% were unmarried and single, 21.90% were unmarried but not single, 13.40% were married without children, and 45.42% were married with children. Regarding educational level, 3.59% had a college degree or lower, 52.29% held a bachelor’s degree, 35.29% held a master’s degree, and 8.82% held a doctoral degree. In terms of work experience, 21.57% had 5 years or fewer, 24.18% had 6–10 years, 27.45% had 11–15 years, 14.38% had 16–20 years, and 12.42% had 21 years or more. Regarding monthly income, 9.15% earned less than 5000 CNY, 36.60% earned between 5000 and 8000 CNY, 32.68% earned between 8000 and 12,000 CNY, and 21.57% earned more than 12,000 CNY.

The results indicated that the five-factor model provided the best fit (*χ*^2^ = 136.937, *df* = 109, SRMR = 0.020, CFI = 0.995, TLI = 0.994, and RMSEA = 0.029). Furthermore, as shown in Table 3, the composite reliability (CR) for each dimension of Generative AI Literacy exceeded 0.7, and the average variance extracted (AVE) values for all dimensions were greater than 0.5, indicating strong convergent validity for the Generative AI Literacy Scale.

To enhance the practical applicability of the scale and improve response efficiency in subsequent research and organizational applications, we recommend constructing a streamlined 10-item short-form scale based on the results of both exploratory factor analysis (EFA) and confirmatory factor analysis (CFA). This abbreviated scale would be developed by selecting the two highest factor-loading items from each of the five dimensions, creating a concise measurement instrument that significantly reduces respondent burden and improves questionnaire completion rates while preserving the theoretical integrity of the five-dimensional GAIL framework.

## 3. Theoretical Basis and Research Hypotheses

### 3.1. Generative AI Literacy and Job Performance

Generative AI Literacy (GAIL), as an essential occupational competence in the digital era, has become a key driver of employee job performance. From the perspective of theoretical development, GAIL can be regarded as an extension and reconstruction of traditional information technology literacy in the age of generative AI. It integrates diverse competencies such as technical operation and critical thinking, forming a composite literacy structure aligned with the demands of the knowledge economy. According to the Ability–Motivation–Opportunity (AMO) framework, employee performance is shaped by the multiplicative interaction of ability, motivation, and opportunity (Appelbaum, 2000; Dasí et al., 2021). The core proposition of the AMO theory posits a multiplicative relationship—rather than an additive one—among these three components, expressed as P = f(A × M × O). This multiplicative formulation implies that all three factors are indispensable and mutually reinforcing. This multiplicative model provides a more comprehensive framework for understanding how GAIL influences job performance through interconnected pathways.

First, GAIL enhances employees’ capabilities by improving their technical proficiency. In the AMO framework, ability is regarded as a fundamental determinant of task performance, with skill mastery directly linked to performance outcomes (Appelbaum, 2000; Demortier et al., 2014). From the perspective of cognitive resource theory, GAIL’s dimensions of basic technical ability, prompt engineering, and content evaluation reduce cognitive load and free up resources for higher-order problem-solving and creative thinking. This optimized cognitive allocation enables employees to perform more efficiently and accurately in complex tasks. Generative AI tools—such as ERNIE Bot, ChatGPT, and DALL·E—facilitate rapid idea generation and problem-solving, reducing time spent on repetitive tasks and unlocking employees’ creative potential (Eapen et al., 2023). Frequent use of such tools allows employees to accumulate practical experience and improve technical fluency, resulting in enhanced performance, especially in high-complexity, knowledge-intensive roles (Haith & Krakauer, 2018). Within the five-dimensional GAIL framework, basic technical ability, prompt engineering, and content evaluation together strengthen the “ability” component, complementing each other to create a comprehensive chain of technical competence. Among these, content evaluation is particularly critical for ensuring the quality and reliability of AI outputs, thereby safeguarding the accuracy and effectiveness of work outcomes.

Second, GAIL can stimulate employees’ intrinsic motivation by increasing their sense of control and accomplishment at work. Motivation is a fundamental psychological force that drives employees to engage actively in their work, and its intensity is influenced by the perceived meaningfulness of the task and the satisfaction derived from its completion (Appelbaum, 2000). According to Self-Determination Theory, intrinsic motivation arises from the fulfillment of three basic psychological needs: autonomy, competence, and relatedness. GAIL’s innovative application ability primarily enhances the “motivation” component by supporting these needs through enhancing employees’ proactivity and creativity. When employees can flexibly use generative AI to support innovative practices, they experience not only technological empowerment but also a heightened sense of personal value and professional identity. Generative AI provides real-time feedback and intelligent suggestions, helping employees solve problems efficiently and gain positive reinforcement, which in turn enhances their sense of task control (Noy & Zhang, 2023). Research indicates that employees with higher levels of AI literacy can derive stronger emotional engagement from these tools, which in turn fosters motivation and work engagement (Brynjolfsson et al., 2023; Keshishi & Hack, 2023). Additionally, GAIL can reduce anxiety and resistance toward new technologies, alleviate technophobia, and promote a more positive attitude and willingness to adopt AI—thus amplifying the motivational impact described by the AMO framework.

Third, GAIL can enhance performance by expanding the “opportunity” component through creating structured spaces for responsible innovation and optimizing collaborative processes. Within the AMO framework, “opportunity” refers to the resources and conditions provided to employees to support their development (Akhtar et al., 2022). GAIL’s dimension of ethical and compliance awareness plays a key role in shaping the opportunity space by establishing clear boundaries for appropriate AI use, thereby creating structured “opportunity spaces” for responsible innovation. While this reduces uncertainty and risk, it may also constrain certain applications when organizations limit AI usage due to privacy or regulatory concerns. Moreover, generative AI’s capabilities in data analysis and forecasting help employees identify cross-functional collaboration opportunities, increasing their sense of participation and influence within the organization. At the organizational level, the diverse competencies encompassed by GAIL foster new models of human–AI collaboration. These models transcend traditional job boundaries, establishing novel interaction mechanisms between humans and machines, and between departments. Such transformations redefine the “opportunity” dimension in the workplace. Research shows that generative AI helps break down information silos and facilitates cross-functional collaboration, thereby enhancing efficiency and performance (Bilgram & Laarmann, 2023).

In sum, GAIL systematically enhances employee performance by strengthening ability, stimulating motivation, and expanding opportunities. From an integrated AMO perspective, the five dimensions of GAIL contribute through multi-pathway mechanisms: basic technical ability, prompt engineering, and content evaluation primarily reinforce “ability”; innovative application ability enhances “motivation”; while ethical and compliance awareness creates structured “opportunity spaces.” This systematic alignment between multidimensional literacy and AMO components constitutes the theoretical mechanism by which GAIL influences job performance. Empirical studies suggest that high AI literacy significantly enhances employees’ Self-Efficacy, thereby improving work efficiency and quality. Therefore, the following hypothesis is proposed:

**Hypothesis** **1:** *Generative AI Literacy has a significant positive effect on job performance.*

### 3.2. Generative AI Literacy and Creative Self-Efficacy

Creative Self-Efficacy (CSE) refers to an individual’s belief in their ability to successfully perform creative tasks and produce innovative outcomes. It is a key psychological driver of innovative behavior (Tierney & Farmer, 2002). Within the AMO theoretical framework, CSE represents a psychological integration that emerges from the interaction of perceived ability, motivational strength, and opportunity recognition. According to AMO theory, efficacy beliefs are shaped by the multiplicative interaction of ability, motivation, and opportunity (Appelbaum, 2000; Blumberg & Pringle, 1982), meaning that all three elements contribute to the development of creative confidence. Generative AI Literacy (GAIL), by enhancing ability, stimulating motivation, and expanding opportunity, can effectively strengthen employees’ Creative Self-Efficacy.

First, GAIL enhances employees’ confidence in creative tasks by improving their technical competence. According to AMO theory, ability is a foundational factor influencing both performance and Self-Efficacy (Kellner et al., 2019). The dimensions of basic technical ability, prompt engineering, and content evaluation in GAIL strengthen the “ability” component of the AMO model. These abilities reduce the barriers to technology use and improve accuracy in quality assessment, enabling employees to experience a stronger sense of competence in innovation tasks, thereby enhancing their Creative Self-Efficacy. Generative AI tools such as ChatGPT and DALL·E support knowledge integration and idea refinement through efficient information processing and real-time feedback, which boost employees’ confidence in their innovative capacity. For example, studies have shown that AI literacy significantly improves employees’ confidence in handling knowledge-intensive tasks (Ng et al., 2024; Putjorn & Putjorn, 2023). Bozkurt (2024b) further noted that generative AI reduces the cost of trial-and-error in creative processes, thus enhancing employees’ confidence and capability in innovation exploration.

AMO theory emphasizes that ability development occurs progressively through accumulated practice. The prompt engineering dimension of GAIL fosters skill development through iterative refinement in real-world tasks, helping employees gradually build stable confidence in their creative abilities—ultimately reinforcing their Creative Self-Efficacy.

Second, GAIL enhances employees’ Creative Self-Efficacy by stimulating intrinsic motivation. Motivation is a core psychological factor in innovation, directly influencing task engagement and self-confidence (Appelbaum, 2000; Saether, 2019). In the AMO framework, motivation serves as the key mechanism through which ability is translated into action. GAIL—especially through its innovative application ability—satisfies employees’ needs for achievement and self-realization, thereby activating strong intrinsic motivation. This motivation, in turn, reinforces employees’ Creative Self-Efficacy through sustained engagement. Generative AI tools provide intelligent suggestions and efficiently generate creative outputs, enhancing employees’ sense of control and accomplishment in completing tasks. For instance, studies have found that real-time feedback and precise support from generative AI significantly enhance employees’ motivation and efficacy under high-pressure environments (Bilgram & Laarmann, 2023). In addition, technology-driven motivation has been shown to positively influence employees’ innovation performance (Li et al., 2020).

AMO theory further highlights that motivation is shaped by both individual traits and contextual conditions. GAIL contributes to a supportive technological environment that sustains motivational engagement. Each successful use of generative AI to solve problems provides positive reinforcement, and this continuous environment-individual interaction significantly enhances Creative Self-Efficacy.

Finally, GAIL increases employees’ Creative Self-Efficacy by providing diversified opportunities for innovative practice. According to AMO theory, opportunity is the critical factor that enables the transformation of ability and motivation into actual performance (Kellner et al., 2019). The “opportunity” component emphasizes external support conditions for individuals to exercise their capabilities. In GAIL, the dimension of ethical and compliance awareness creates structured “opportunity spaces” by establishing clear boundaries for appropriate AI use. This structured and regulated space reduces uncertainty and risk, providing employees with a secure environment to explore generative AI’s creative potential—ultimately reinforcing their Creative Self-Efficacy. Generative AI offers diverse applications such as cross-department collaboration, creative validation, and data analysis, enabling employees to better unleash their creative capacities. Studies show that generative AI expands the innovation frontier and optimizes the work environment, thereby creating more opportunities for idea implementation and significantly enhancing employee innovation outcomes (Mukherjee & Chang, 2023).

Within the AMO framework, opportunity also includes resource availability and organizational support. GAIL’s prompt engineering ability increases the efficiency of resource utilization, maximizing the creative output from limited AI resources. Meanwhile, the ethical and compliance awareness dimension reduces organizational resistance and strengthens institutional support for AI-driven innovation. These two aspects jointly enhance the “opportunity” component and provide strong support for the formation of Creative Self-Efficacy.

In summary, GAIL enhances employees’ Creative Self-Efficacy through the combined effects of improving ability, stimulating motivation, and expanding opportunity. From the integrated AMO perspective, GAIL’s five dimensions contribute through interconnected mechanisms: basic technical ability, prompt engineering, and content evaluation reinforce ability acquisition; innovative application ability fuels motivational engagement; while ethical compliance awareness creates developmental opportunities. These pathways are mutually reinforcing within the multiplicative AMO structure (P = f(A × M × O)), jointly constructing a complete theoretical chain of “Ability–Motivation–Opportunity–Self-Efficacy.” This multi-pathway model grounded in AMO theory offers a more comprehensive explanation of how technological literacy translates into psychological efficacy. Beyond enhancing individuals’ confidence and performance in creative tasks, it also provides theoretical foundations and practical insights for organizations aiming to boost overall innovation capacity. Accordingly, the following hypothesis is proposed:

**Hypothesis** **2:** *Generative AI Literacy has a significant positive effect on Creative Self-Efficacy.*

### 3.3. Creative Self-Efficacy and Job Performance

Creative Self-Efficacy (CSE) is one of the most critical psychological factors influencing individual job performance. It enhances performance by activating behavioral motivation, strengthening problem-solving capabilities, and facilitating resource integration (Tierney & Farmer, 2002; Q. Zhou et al., 2012; Newman et al., 2018). Within the overarching structure of the AMO framework, CSE functions as a psychological mechanism that affects performance through multiple pathways: it optimizes the effective deployment of abilities, reinforces the intensity and persistence of motivation, and enhances the recognition and utilization of opportunities. Under the AMO model, CSE plays a significant role in boosting individual capability and motivation, especially in the context of complex tasks (Cui & Yu, 2021; Tierney & Farmer, 2002).

First, from the AMO perspective, high levels of Creative Self-Efficacy enhance employees’ performance in innovation-oriented tasks (Cui & Yu, 2021). The “ability” dimension of the AMO framework encompasses not only objective skills but also the effective activation and integration of these skills. CSE enhances employees’ capacity to draw upon their knowledge and skill reserves when confronting complex challenges, thereby reducing underutilization and fragmentation of capability. CSE fosters confidence in dealing with complex tasks, enabling individuals to solve problems efficiently through innovative thinking and resource coordination (Haase et al., 2018). Empirical research has shown that employees with high CSE are more likely to embrace challenging tasks and demonstrate higher levels of proactivity and problem-solving ability, which directly contribute to enhanced organizational performance (Newman et al., 2018). Moreover, CSE increases employees’ identification with organizational goals, further motivating their engagement and contributions to creative endeavors (Orth & Volmer, 2017).

CSE also contributes to job performance by transforming the “motivation” (M) dimension of the AMO framework into sustained action. According to AMO theory, the strength and persistence of motivation are key determinants of performance outcomes. CSE enhances employees’ resilience and perseverance when facing innovation challenges, making them more likely to persist rather than withdraw when encountering obstacles. This persistence significantly increases the likelihood and quality of task completion. Furthermore, CSE facilitates resource integration and collaboration, particularly in innovation tasks. Employees with high CSE can effectively leverage technological tools to optimize task execution and promote efficient resource coordination through cross-functional collaboration (Orth & Volmer, 2017; Namono et al., 2022). This capability not only improves individual performance but also contributes to broader organizational innovation. Studies confirm that employees with high CSE demonstrate higher efficiency and output quality in complex tasks compared to those with lower CSE, affirming the positive relationship between CSE and job performance (Kong et al., 2019).

From the AMO perspective, CSE also enhances job performance by expanding the “opportunity” (O) dimension. Employees with high Creative Self-Efficacy are more likely to proactively identify and seize innovation opportunities rather than passively waiting for them. Such opportunity-seeking and opportunity-creation behaviors allow them to discover and utilize more resources and support within the same objective environment, effectively enlarging the “opportunity space” defined in the AMO model.

Moreover, Generative AI Literacy (GAIL) further enhances CSE through technological empowerment, which subsequently contributes to improved job performance. Specifically, GAIL enables employees to better apply technological tools in innovation tasks, thereby increasing their confidence and competence in task execution. Generative AI tools not only offer real-time feedback and intelligent suggestions but also reduce psychological stress and enhance employees’ sense of control over innovation tasks (Bilgram & Laarmann, 2023; Wang et al., 2023). This process of technological empowerment reinforces the interactive dynamics of the AMO model: technology boosts ability, successful experiences enhance motivation, and innovative applications expand opportunity. These three elements work synergistically within the multiplicative framework (P = f(A × M × O)) to form a positive, upward spiral. Such technological support enables high-CSE employees to integrate resources more effectively and foster cross-departmental collaboration, further enhancing organizational performance.

It is important to note that from the AMO perspective, the impact of CSE on job performance is context dependent. In work environments characterized by high task complexity, strong innovation demands, and adequate resource availability, the influence of CSE on performance is particularly significant. This is because such environments place higher demands on the alignment and optimization of the AMO triad, and CSE is uniquely capable of sustaining ability and motivation while creatively expanding the boundaries of opportunity.

In summary, Creative Self-Efficacy enhances individual innovation performance and job outcomes by strengthening innovation confidence, facilitating resource integration, and promoting cross-functional collaboration. A systematic analysis based on the AMO framework reveals that CSE serves as a psychological integrator that aligns ability, motivation, and opportunity toward performance outcomes through the multiplicative dynamics of the model. This multidimensional influence mechanism provides a more comprehensive understanding of CSE’s value in organizational settings. Accordingly, the following hypothesis is proposed:

**Hypothesis** **3:** *Creative Self-Efficacy has a significant positive effect on job performance.*

### 3.4. The Mediating Effect of Creative Self-Efficacy

According to the AMO framework, employee job performance is shaped by the synergistic interaction of ability, motivation, and opportunity, with mediating variables serving as critical psychological bridges in this process (Edgar et al., 2021; Nadeem & Rahat, 2021). The AMO model emphasizes that ability (A), motivation (M), and opportunity (O) interact through complex conversion mechanisms characterized by multiplicative rather than additive relationships. This multiplicative nature (P = f(A × M × O)) means that mediating variables can amplify effects across multiple AMO dimensions simultaneously. As a key psychological conduit between Generative AI Literacy (GAIL) and job performance, Creative Self-Efficacy (CSE) enhances innovation confidence and optimizes task execution pathways, thereby converting technological capabilities into tangible performance outcomes (Kong et al., 2019).

GAIL promotes the development of CSE by strengthening employees’ technical competencies and supporting innovation. From the AMO perspective, GAIL enhances the “ability” dimension, thereby providing an objective foundation for the formation of CSE. Compared to general skills, GAIL particularly enhances algorithmic thinking and the ability to apply intelligent tools—skills that are highly aligned with the innovation demands of modern work environments. Consequently, these capabilities are more effectively transformed into Creative Self-Efficacy. Specifically, GAIL offers efficient data processing and real-time decision-making support through AI tools, empowering employees to face complex tasks with greater innovation confidence (Brynjolfsson et al., 2023). Additionally, the instant feedback and intelligent suggestions provided by generative AI improve task efficiency and the visibility of results, thereby enhancing employees’ sense of achievement and intrinsic motivation (Ebert & Louridas, 2023).

Within the AMO framework, CSE serves as a key psychological mechanism that transforms “ability” (A) into “motivation” (M). According to AMO theory, ability alone does not automatically result in action—it must be activated by motivation. CSE, as a form of efficacy belief, serves precisely this purpose: it translates the technological capacity conferred by GAIL into innovative motivation, shifting employees from “can do” to “want to do.” This transformation process operates through the interconnected pathways established by GAIL’s five dimensions: basic technical ability, prompt engineering, and content evaluation build foundational confidence; innovative application ability directly enhances creative motivation; while ethical compliance awareness creates structured opportunity spaces. Employees with high CSE are more likely to leverage generative AI tools to develop creative solutions and facilitate resource integration and implementation through cross-functional collaboration.

Furthermore, CSE facilitates the transformation of GAIL into job performance by expanding the “opportunity” (O) dimension. AMO theory posits that opportunity is a necessary condition for ability and motivation to be translated into performance outcomes. CSE enhances employees’ proactiveness in recognizing, seizing, and creating opportunities, enabling them to identify more resource support and application scenarios within the organizational environment. As a result, GAIL can be more effectively applied in practical work contexts to produce meaningful outcomes. This process of opportunity recognition and creation effectively broadens the “opportunity space” defined within the AMO model. Empirical studies have found that under conditions of resource constraints and high task complexity, CSE plays an even more critical role, exhibiting a strong amplifying effect on performance (Haase et al., 2018; Kong et al., 2019), thereby further confirming its mediating influence.

Given the multiplicative nature of the AMO model—P = f(A × M × O)—CSE, as a mediating variable, exerts its influence across all three components, meaning its mediating effect is amplified through multiplicative interactions rather than simple additive effects. This multiplicative mediation helps explain why GAIL, when acting through CSE, often has a disproportionately strong effect on performance outcomes. Moreover, CSE strengthens employees’ positive reinforcement loops by promoting a “belief–action–outcome” cycle (Farmer & Tierney, 2017). This cycle not only drives individual performance improvement but also provides sustained support for organizational innovation capabilities.

The AMO framework places particular emphasis on contextual factors affecting the realization of capabilities. In today’s rapidly evolving work environments—where generative AI technologies are constantly updating—CSE plays an adaptive role. Employees with high CSE exhibit greater learning agility and adaptability in the face of technological change, allowing GAIL to remain an effective driver of performance in dynamic environments and demonstrating the temporal stability of CSE’s mediating role.

In summary, organizations can enhance overall performance by cultivating employees’ Generative AI Literacy and, through it, strengthening their Creative Self-Efficacy (Rukadikar & Khandelwal, 2023; Shafiabady et al., 2023). Based on a systematic analysis using the AMO framework, CSE functions as a psychological mediator between GAIL and job performance through a comprehensive mechanism: it transforms GAIL’s technical ability into innovative motivation, activating the M dimension; it fosters opportunity identification and creation, expanding the O dimension; and it harmonizes the interaction among ability, motivation, and opportunity to maximize their multiplicative effect. This comprehensive mediating mechanism illustrates the internal pathway by which technological capabilities are converted into performance outcomes through enhanced Creative Self-Efficacy. The synergy between technological capability enhancement and creative confidence development not only enhances individual innovation and work performance but also lays a solid foundation for sustained organizational innovation. Therefore, the following hypothesis is proposed:

**Hypothesis** **4:** *Creative Self-Efficacy partially mediates the relationship between Generative AI Literacy and job performance.*

**Figure 1:**
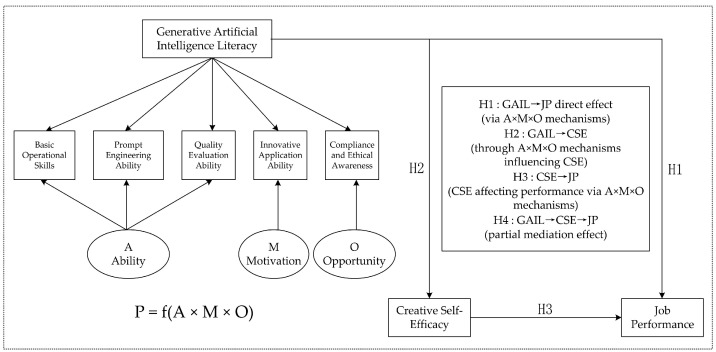
Integrated Theoretical Model: GAIL → CSE → Job Performance.

The theoretical model and hypothesized relationships are illustrated in Figure 1.

## 4. Research Design

### 4.1. Research Context and Design

This empirical validation study was conducted in mainland China between September and November 2024, following the completion of scale development phases that established the psychometric properties of the Generative AI Literacy (GAIL) scale through exploratory and confirmatory factor analyses using private sector data. The empirical phase leveraged China’s rapid advancement in digital transformation and the widespread organizational adoption of generative AI technologies to provide a rich context for testing the theoretical relationships between GAIL, Creative Self-Efficacy, and job performance.

The empirical validation phase strategically focused on public sector employees to examine the cross-contextual applicability of the GAIL scale and validate the hypothesized theoretical relationships in a context where generative AI adoption has reached significant scale. This approach enhances the generalizability of our findings by demonstrating that both the GAIL measurement framework and the underlying theoretical model hold across different organizational environments from those used in scale development.

The strategic focus on public sector employees in the empirical validation is justified by several contextual factors specific to China’s organizational landscape. First, the public sector has emerged as the primary domain for large-scale generative AI implementation in China. Under the national digital government strategy, public sector organizations have become the most significant adopters of generative AI technologies, with extensive secondary data consistently indicating that state-owned enterprises and central government entities are the primary purchasers of AI large language models, with implementation depth and breadth significantly exceeding private sector adoption. Specifically, by early 2025, over 200 public sector organizations at different administrative levels had completed local deployment of large language models, with more than ten municipal governments in the Yangtze River Delta region alone announcing integration with domestic large language models. The scale and scope of this deployment is evidenced by practical outcomes: Shenzhen Futian District’s “AI Digital Employee” system achieved over 95% formatting accuracy and reduced review time by 90%; personalized policy matching systems demonstrated identification accuracy exceeding 90%; and Nanjing’s “Ningan Qing” emergency management large language model improved overall administrative efficiency by approximately 70%. However, this rapid technological deployment has revealed a significant capability gap among public sector employees. Survey data from 1336 personnel indicates that while 84.6% express interest in “AI-enabled government services,” only approximately one-third can proficiently master and appropriately apply core generative AI functions. Furthermore, 91.73% of respondents express concerns about data privacy breaches, 65.35% worry about diminished human judgment, and 61.42% fear data fabrication risks, highlighting the critical need for comprehensive AI literacy development in public sector contexts.

Second, China’s public sector encompasses a distinctive institutional structure that includes not only traditional government agencies and public institutions but also the extensive state-owned enterprise system, which together constitute the core infrastructure for national governance and public service delivery. This institutional breadth is supported by strategic policy initiatives, including the inclusion of “AI+” action plans in the 2024 Government Work Report and the designation of “AI+” initiatives as one of nine priority tasks for 2025 by the Central Economic Work Conference, demonstrating the government’s commitment to AI integration across public sector operations.

Third, public sector employees’ AI literacy development is directly linked to China’s national governance modernization objectives, as their generative AI competencies directly influence the transformation of technological capabilities into effective governance outcomes and public service quality. This relationship is particularly evident in international comparative context, where countries such as the United States have implemented specialized training programs for public sector employees on responsible generative AI use through initiatives like InnovateUS, and the United Kingdom has released the “AI Playbook for Government” to guide safe and effective AI implementation across government departments. The UN E-Government Survey 2024 emphasizes that while AI provides significant opportunities for enhancing public sector efficiency, systematic investment in AI literacy development is essential to prevent the exacerbation of global digital divides, underscoring the strategic importance of comprehensive AI literacy frameworks for public sector employees.

### 4.2. Sample Selection and Data Collection Procedures

To examine the hypothesized theoretical relationships and validate the cross-contextual applicability of the GAIL scale, data were collected from employees working in the Chinese public sector for the empirical validation phase. Public sector participants were identified through the following organizational classification: respondents selected their current workplace as (1) state-owned enterprise/state-holding enterprise, (2) public institution, or (3) government agency from a comprehensive organizational type questionnaire.

Two sampling methods were adopted for participant recruitment: (1) snowball sampling through the researchers’ social networks, including classmates, colleagues, and friends in public sector organizations; and (2) online distribution via the Wenjuanxing platform, targeting a broader range of public sector employees across different government levels and state-owned enterprises.

Consistent with standard research ethics, participants were informed of this study’s purpose and assured of anonymity and data confidentiality. To minimize common method bias and enhance causal inference validity, a multi-wave survey design was employed. Data collection occurred in three sequential phases: Phase 1 collected GAIL scale responses and demographic information (423 responses); Phase 2 collected Creative Self-Efficacy responses (397 responses); Phase 3 collected Job Performance responses (372 responses). Response rates were 93.9% (Wave 1–2) and 93.7% (Wave 2–3), with an overall completion rate of 87.9%. After data screening, 344 valid questionnaires remained for analysis.

### 4.3. Sample Characteristics

The final sample of 344 valid respondents from the Chinese public sector demonstrated diverse demographic characteristics that reflect the composition of China’s modern public sector workforce.

Gender and Age Distribution. Regarding gender distribution, 49.71% of the respondents were male, and 50.29% were female, indicating a balanced gender representation. In terms of age, 20.93% were aged 25 and below, 40.41% were aged 26–35, 25.87% were aged 36–45, 10.76% were aged 46–55, and 2.03% were aged 56 and above. The age distribution shows that the majority of participants (66.34%) were in the 26–45 age range, representing the core working population in China’s public sector.

Marital Status and Family Structure. For marital status, 20.64% were single, 22.38% were in a relationship but unmarried, 14.83% were married without children, and 42.16% were married with children. This distribution reflects the family structure patterns typical of Chinese public sector employees, with the majority (56.99%) being married.

Educational Background. In terms of education level, 2.91% held an associate degree or below, 55.81% had a bachelor’s degree, 33.14% held a master’s degree, and 8.14% held a doctoral degree. The high educational attainment is notable, with 96.09% of participants holding bachelor’s degrees or higher, and 41.28% possessing advanced degrees (master’s or doctoral), reflecting the educational requirements and professional development emphasis within China’s public sector organizations.

Professional Experience. Regarding work experience, 22.67% had worked for 5 years or less, 23.26% for 6–10 years, 27.91% for 11–15 years, 13.95% for 16–20 years, and 12.21% for over 21 years. This distribution indicates a balanced representation across different career stages, with the largest group (27.91%) having 11–15 years of experience, suggesting a mature and experienced workforce.

Income Distribution. Monthly income levels were distributed as follows: 20.64% earned below 5000 RMB, 42.44% earned between 5000 and 8000 RMB, 31.10% earned between 8000 and 12,000 RMB, and 5.81% earned above 12,000 RMB. The income distribution reflects the salary structure of China’s public sector, with the majority (73.54%) earning between 5000 and 12,000 RMB monthly, which aligns with the compensation levels for professional positions in Chinese government institutions and state-owned enterprises.

Sample Representativeness. This demographic profile demonstrates that the sample adequately represents the diversity of China’s public sector workforce across key characteristics including age, education, experience, and organizational levels. The high educational attainment and professional experience levels of participants provide a solid foundation for examining Generative AI Literacy in contexts where such technologies are increasingly being deployed for governance and public service delivery.

### 4.4. Variable Measurement

Generative Artificial Intelligence Literacy. The measurement of this construct was based on a self-developed scale. The scale consists of five dimensions and 17 items, using a 7-point Likert scale (1 = strongly disagree, 7 = strongly agree). The scale is designed to assess individuals’ overall literacy in the use of generative artificial intelligence technologies. Example items include: “I can solve common technical issues during the use of AI.” In this study, Cronbach’s α coefficient of the scale was 0.943, indicating good internal consistency.

Creative Self-Efficacy. The measurement of Creative Self-Efficacy was based on the scale developed by Carmeli and Schaubroeck (2007), consisting of 8 items using a 6-point Likert scale (1 = strongly disagree, 6 = strongly agree). This scale assesses employees’ confidence and belief in their ability to generate creative solutions and produce innovative outcomes in work contexts. Example items include: “I can creatively overcome many challenges”. In this study, Cronbach’s α coefficient was 0.916, indicating high reliability.

Job Performance. The measurement of job performance was based on the scale developed by Chen et al. (2002), consisting of 4 items using a 7-point Likert scale (1 = strongly disagree, 7 = strongly agree). This scale measures employees’ actual behavioral contributions and task completion effectiveness within their organizational role, which conceptually differs from Creative Self-Efficacy in several key ways. While Creative Self-Efficacy captures confidence and belief in one’s creative abilities, job performance measures observable work behaviors and outcomes such as task completion, quality standards, and organizational contributions. Example items include “I can complete the tasks assigned by the department on time”. In this study, Cronbach’s α coefficient was 0.885, indicating good internal consistency.

Control Variables. To improve the rigor of this study, the research included several control variables, such as employees’ gender, age, education level, work experience, and monthly income (Zhao et al., 2022; Wang et al., 2023; Baabdullah, 2024). Additionally, variables related to employee characteristics, such as job type, job rank, and political affiliation, were also controlled for (Pinski et al., 2024). The selection of these control variables helps to further reduce the potential influence of confounding factors on this study’s results.

### 4.5. Statistical Analysis

This study employed SPSS27, Amos24, and R-4.3.3 software to conduct comprehensive statistical analyses following a systematic approach. Exploratory factor analysis (EFA) was performed using SPSS, followed by confirmatory factor analysis (CFA) and reliability testing using Amos to ensure adequate measurement model properties. The primary hypothesis testing was conducted using R software through a two-stage analytical strategy: first, hierarchical regression analysis was employed to examine direct relationships between key variables (GAIL → CSE, GAIL → Job Performance, CSE → Job Performance) while controlling for demographic and organizational factors; second, mediation effect analysis was conducted using bootstrap methodology with 5000 resamples to test indirect effects and examine the mediating role of Creative Self-Efficacy. This analytical sequence follows established best practices where direct relationships are established through regression analysis, followed by bootstrap procedures for mediation testing, which provides robust confidence intervals for indirect effects without requiring normality assumptions.

## 5. Research Results

### 5.1. The Common Method Bias Test

To minimize the impact of potential common method bias, this study implemented several measures from both procedural and statistical controls. In terms of procedural control, this study used anonymous surveys and explicitly assured participants that their data would be kept strictly confidential to reduce concerns and enhance the authenticity of their responses. Additionally, the questionnaire design prioritized simplicity and readability, ensuring that the questions were clear and easy to understand. Furthermore, this study adopted a multi-wave data collection approach to reduce the possibility of common method bias from a temporal perspective. In terms of statistical control, this study assessed common method bias using Harman’s single-factor test. The results showed that the variance explained by the first factor was 21.719%, which is below the 50% threshold. This indicates that the common method bias in the data of this study is within acceptable limits.

### 5.2. Reliability and Validity Testing

This study rigorously evaluated the reliability and validity of the scales to ensure the reliability and validity of the measurement tools. Reliability analysis included Cronbach’s α coefficient and composite reliability (CR), while validity was assessed through average variance extracted (AVE). The results are shown in Table 4. In terms of reliability, Cronbach’s α values for Generative AI Literacy, Creative Self-Efficacy, and Job Performance were 0.943, 0.916, and 0.885, respectively. The composite reliability (CR) values for these scales were 0.868, 0.917, and 0.885, all of which significantly exceed the recommended threshold of 0.7. This indicates good internal consistency and high reliability for all the scales. In terms of validity, the average variance extracted (AVE) values for the scales were 0.564, 0.579, and 0.658, all above the 0.5 standard. This indicates that the items of the scales adequately reflect the latent constructs they measure, and the convergent validity meets the required standards.

### 5.3. Confirmatory Factor Analysis of the Model

This study conducted a confirmatory factor analysis (CFA) on the data using R software, with the results shown in Table 5. Model comparison indicates that the three-factor model (Generative Artificial Intelligence Literacy, Creative Self-Efficacy, and Job Performance) provides the best fit, with the following indices: *χ*^2^ = 402.963, *df* = 369, CFI = 0.996, TLI = 0.995, SRMR = 0.033, and RMSEA = 0.016, all meeting high fit standards. In comparison, the two-factor model showed slightly worse fit indices than the three-factor model (*χ*^2^ = 621.869, *df* = 371, CFI = 0.968, TLI = 0.965, SRMR = 0.074, and RMSEA = 0.044). The one-factor model showed the poorest fit (*χ*^2^ = 739.038, *df* = 372, CFI = 0.954, TLI = 0.950, SRMR = 0.071, and RMSEA = 0.054). The results indicate that the three-factor model significantly outperforms the two-factor and one-factor models, demonstrating good discriminant validity among Generative Artificial Intelligence Literacy (GAIL), Creative Self-Efficacy (CSE), and Job Performance (JP) in this study.

### 5.4. Descriptive Statistics and Correlation Analysis

The means, standard deviations, and correlation coefficients of the variables are presented in Table 6. As shown in Table 6, Generative AI Literacy is significantly positively correlated with Creative Self-Efficacy (r = 0.661, *p* < 0.001), Generative AI Literacy is significantly positively correlated with Job Performance (r = 0.706, *p* < 0.001), and Creative Self-Efficacy is significantly positively correlated with Job Performance (r = 0.742, *p* < 0.001). Given that the correlation coefficients exceed 0.7, collinearity tests were further conducted in this study. The results showed that the Variance Inflation Factors (VIF) for all variables were below 5, indicating that there is no severe multicollinearity issue among the variables.

### 5.5. Hypothesis Testing

This section examines the mechanism through which Generative Artificial Intelligence Literacy influences job performance and tests the mediating role of Creative Self-Efficacy between the two. The specific results are presented in Table 7. First, Creative Self-Efficacy was set as the outcome variable, and Models 1 and 2 were constructed. Model 1 includes only the control variables to analyze the effect of the control variables on Creative Self-Efficacy. Model 2 builds upon Model 1 by adding Generative AI Literacy to explore the direct effect of Generative AI Literacy on Creative Self-Efficacy. Next, Job Performance was set as the outcome variable, and Models 3 through 6 were constructed. Model 3 includes only the control variables to analyze their independent effect on Job Performance. Model 4 builds upon Model 3 by adding Generative AI Literacy to test its direct effect on Job Performance. Model 5 adds Creative Self-Efficacy to Model 3 to analyze its effect on Job Performance. Model 6 builds upon Model 4 by adding both Generative AI Literacy and Creative Self-Efficacy to further explore the direct effect of Generative AI Literacy on Job Performance and the mediating role of Creative Self-Efficacy.

According to the analysis results, Model 2 shows that Generative AI Literacy has a significant positive effect on Creative Self-Efficacy (*β* = 0.640, *p* < 0.001), supporting Hypothesis 2. Model 4 indicates that the direct effect of Generative AI Literacy on Job Performance is significant (*β* = 0.680, *p* < 0.001), supporting Hypothesis 1. Model 5 shows that Creative Self-Efficacy has a significant positive effect on Job Performance (*β* = 0.713, *p* < 0.001), supporting Hypothesis 3. The analysis of Model 6 further demonstrates that when both Generative AI Literacy and Creative Self-Efficacy are included, the direct effect of Generative AI Literacy on Job Performance remains significant (*β* = 0.554, *p* < 0.001), and the effect of Creative Self-Efficacy on Job Performance is also significant (*β* = 0.320, *p* < 0.01). This indicates that Creative Self-Efficacy partially mediates the relationship between Generative AI Literacy and Job Performance, thus supporting Hypothesis 4.

This study further employed the Bootstrap method to test the mediating role of Creative Self-Efficacy in the relationship between Generative AI Literacy and Job Performance. By performing 5000 bootstrap resamples and calculating the 95% confidence interval, the results are shown in Table 8. First, the total effect of Generative AI Literacy on Job Performance was 0.680, with a standard error of 0.099, and the confidence interval did not include 0, indicating that the total effect was significant. Secondly, the direct effect of Generative AI Literacy on Job Performance was 0.365, with a standard error of 0.073, and the confidence interval also did not include 0, suggesting that the direct effect of Generative AI Literacy on Job Performance was significant. Finally, the indirect effect of “Generative AI Literacy → Creative Self-Efficacy → Job Performance” was 0.537, with a standard error of 0.132, and the confidence interval did not include 0, indicating that the indirect effect was significant. The results show that Creative Self-Efficacy plays an important mediating role in the relationship between Generative AI Literacy and Job Performance.

## 6. Discussion of Results

This study developed a Generative AI Literacy measurement framework suitable for the workplace through a rigorous scale development process, including item generation, exploratory factor analysis (EFA), and confirmatory factor analysis (CFA). This framework lays the foundation for subsequent theoretical research and practical validation. Based on the Ability–Motivation–Opportunity (AMO) theory, this study further explored the mechanism through which Generative AI Literacy (GAIL) influences job performance and tested the mediating effect of Creative Self-Efficacy. Through empirical analysis of 344 samples from the public sector, the results show that Generative AI Literacy has a significant positive effect on employees’ job performance, and Creative Self-Efficacy plays a partial mediating role in the relationship between Generative AI Literacy and job performance.

### 6.1. Theoretical Contributions

First, this study advances the theoretical understanding of Generative AI Literacy (GAIL) by developing a comprehensive, workplace-oriented measurement framework that addresses critical gaps in the existing literature. While previous research has predominantly focused on educational contexts or platform-specific applications (Ng et al., 2024; Lee & Park, 2024), this study constructs a theoretically grounded yet practically relevant framework specifically designed for diverse professional environments. The five-dimensional structure—encompassing basic technical ability, prompt engineering, content evaluation, innovative application, and ethical compliance—represents an integrated competency system where technical proficiency, creative application, and ethical awareness work synergistically to enable effective human–AI collaboration across various organizational contexts.

Our methodological approach of developing the scale using private sector data while validating theoretical relationships in public sector contexts demonstrates the framework’s cross-sectoral applicability, providing evidence that GAIL captures fundamental competencies required for effective generative AI use regardless of specific organizational characteristics. Unlike previous models that often treat AI literacy as discrete skills, our framework conceptualizes GAIL as a progressive competence structure that moves from basic technical mastery to innovative application, with ethical awareness serving as an overarching governance mechanism throughout the entire literacy development process.

Second, this study extends the Ability–Motivation–Opportunity (AMO) framework into the emerging domain of generative AI, revealing how technological competencies influence job performance through multiplicative rather than additive pathways. Rather than treating AI literacy as simply another technical skill, our research demonstrates how GAIL operates through the dynamic interactions of the AMO model—simultaneously enhancing employee capabilities, stimulating intrinsic motivation for innovation, and creating structured opportunities for creative technology application. The systematic alignment between GAIL dimensions and AMO components provides theoretical insight into why certain employees derive greater performance benefits from AI technologies than others.

Importantly, our identification of Creative Self-Efficacy as a key mediating mechanism reveals that the pathway from technological competence to performance enhancement operates through enhanced creative confidence rather than direct skill application alone. This finding suggests that effective AI literacy development must address both technical proficiency and psychological readiness for creative technology use, enriching the AMO framework by demonstrating how enhanced creative confidence and innovation-related Self-Efficacy serve as critical pathways through which technical capabilities translate into performance outcomes.

Third, this study contributes to innovation theory by demonstrating how human–AI collaboration can augment rather than replace creative processes. Our findings challenge conventional narratives that position AI as either a threat to human creativity or merely a passive tool, instead supporting an “AI-augmented creativity” perspective where generative AI technologies serve as catalysts that enhance human creative capacity through structured interaction and iterative refinement. The innovative application dimension of GAIL particularly illustrates how employees can leverage AI capabilities to expand their creative boundaries while maintaining human agency in the creative process.

However, our research also reveals significant implementation challenges that organizations must carefully navigate when developing GAIL programs. The integration of technical capabilities with ethical compliance can create tensions between efficiency maximization and regulatory adherence. Our findings suggest that employees may experience conflicts between leveraging AI’s full potential and adhering to organizational guidelines, particularly when ethical considerations appear to constrain applications that could enhance performance (Chaves-Maza & Fedriani, 2025). Furthermore, the rapid evolution of generative AI technologies creates ongoing pressures for continuous learning and adaptation, which may generate resource demands and adaptation stress for both individuals and organizations.

Additionally, while the ethical and compliance dimension is crucial for responsible AI use, our research indicates that this dimension may inadvertently create barriers to innovation if not carefully balanced with performance objectives. Organizations may face challenges in fostering AI experimentation while maintaining ethical standards, potentially leading to overly conservative approaches that limit the innovative potential of generative AI technologies. These implementation realities suggest that while GAIL offers significant performance benefits, successful adoption requires sophisticated organizational change management that balances innovation encouragement with responsible use guidelines.

Fourth, regarding the generalizability and contextual specificity of our findings, this study offers insights that span both universal principles and context-dependent applications. The core theoretical relationships identified in our GAIL-Creative Self-Efficacy-performance model likely represent universal mechanisms grounded in established psychological theories of Self-Efficacy and well-validated organizational frameworks like AMO, suggesting broad applicability across diverse cultural and organizational contexts. The five-dimensional structure of GAIL appears to capture fundamental competencies required for effective human–AI collaboration that transcend specific organizational or cultural boundaries.

However, certain aspects of our findings reflect characteristics specific to our research context that may limit direct generalization. The particularly strong emphasis on ethical and compliance awareness observed in our Chinese public sector sample may be amplified by the regulatory environment and organizational culture specific to Chinese governmental institutions. Similarly, the specific pathways through which GAIL influences performance, and the relative importance of different dimensions, may vary across cultural contexts with different levels of technology acceptance, hierarchical structures, and innovation orientations.

To enhance international applicability, future research should examine these relationships across diverse cultural and organizational contexts to distinguish universal mechanisms from culture-specific applications. This approach would enable researchers and practitioners to identify which aspects of the GAIL framework can be directly applied internationally and which elements require cultural adaptation, thereby maximizing the framework’s contribution to global understanding of AI literacy in organizational contexts.

### 6.2. Managerial Implications

First, establish a scenario-based development system for Generative AI Literacy to facilitate the integration of technical skills with practical experience. The widespread adoption of Generative AI (GAI) technology necessitates that organizations design systematic literacy enhancement programs from both technological and business perspectives. Organizations should develop context-specific “scenario-driven” training systems that incorporate GAI tools into real-world business scenarios relevant to their particular industry and organizational culture. For example, integrating GAI technologies into text generation, data analysis, or customer interactions will enable employees to master the technology through actual tasks. Additionally, targeted modular training courses—covering key areas such as prompt optimization and critical evaluation of generated content—should be developed to ensure comprehensive coverage of the core competencies of Generative AI Literacy (GAIL).

To enhance cross-organizational applicability, organizations should adapt the GAIL framework to their specific contexts while maintaining the core five-dimensional structure. For instance, technology-intensive organizations may emphasize prompt engineering and content evaluation capabilities, while service-oriented organizations might prioritize innovative application and ethical compliance dimensions. Organizations can introduce AI mentors or blended learning platforms, fostering dynamic evaluation and feedback systems to sustain employees’ literacy growth. Furthermore, organizations should avoid the “toolification” trend in technical training, instead emphasizing the integration of technology with creativity, decision making, and organizational values.

Second, strengthen the creative confidence development function of the organizational environment to cultivate innovation-centered culture. Research has found that Creative Self-Efficacy (CSE) plays a key mediating role in how Generative AI Literacy influences job performance. Therefore, organizations should focus on enhancing employees’ Creative Self-Efficacy and innovation confidence through targeted psychological support mechanisms. Organizations can establish innovation labs or technology sandboxes to provide employees with low-risk experimental environments, allowing them to explore technology without fear of failure. Regular “innovation challenges” or cross-departmental collaboration projects can be organized to encourage employees to apply GAI technologies to solve real-world problems, thereby boosting their creative competence beliefs and innovation-related Self-Efficacy.

To further enhance creative confidence development, leaders should actively motivate employees through inspiring communication, public recognition, and constructive feedback, while helping them understand the meaningful impact of their innovative work. This approach stimulates intrinsic motivation through technological empowerment that builds Creative Self-Efficacy rather than creating dependency on AI systems.

Third, develop adaptable resource allocation and policy support systems that can be tailored to diverse organizational contexts. Effective resource allocation and policy support are fundamental to successful GAIL implementation across different organizational environments. Organizations should recognize that GAIL requirements may vary significantly across industries, cultural contexts, and organizational structures, necessitating flexible implementation strategies. First, organizations need to increase investment in Generative AI infrastructure, including hardware upgrades and software optimization, while ensuring that these investments align with their specific operational needs and cultural values.

For organizations operating in culturally diverse environments, particular attention should be paid to adapting ethical guidelines and compliance standards to local regulatory requirements and cultural norms. Cross-departmental resource-sharing platforms should be established, enabling employees to access information resources and technical support through collaborative systems that respect organizational hierarchy and communication patterns. Organizations should establish context-appropriate guidelines for technology application and ethical standards, recognizing that ethical considerations may vary across cultural and regulatory environments.

Fourth, implement adaptive change management strategies that address the implementation challenges identified in our research. Organizations must proactively address potential resistance to AI adoption, substantial training resource requirements, and the ongoing pressures created by rapidly evolving AI technologies. This includes developing comprehensive communication strategies that address employee concerns about job security and technological displacement, while emphasizing the augmentative rather than replacement role of AI technologies.

Organizations should also establish balanced governance frameworks that encourage AI experimentation while maintaining ethical standards and performance accountability. This may involve creating innovation policies that provide clear boundaries for acceptable AI use while encouraging creative exploration, and developing support mechanisms that help employees navigate the tensions between efficiency maximization and ethical compliance.

Finally, to enhance the cross-cultural and cross-organizational applicability of GAIL development programs, organizations should establish systematic adaptation protocols. These protocols should include assessment of local cultural values, regulatory environments, and organizational structures to determine appropriate modifications to the five-dimensional GAIL framework. Organizations operating across multiple cultural contexts should develop culturally sensitive training materials and evaluation criteria while maintaining the core theoretical integrity of the GAIL construct. This adaptive approach enables organizations to leverage the universal benefits of AI literacy development while respecting local contexts and constraints, thereby maximizing both effectiveness and cultural appropriateness.

### 6.3. Limitations and Future Directions

First, while the Generative AI Literacy (GAIL) measurement framework developed in this study has demonstrated strong reliability and validity through rigorous scale development processes, several methodological limitations warrant acknowledgment. Our reliance on self-reported measures, while theoretically justified and methodologically necessary for studying complex psychological constructs like AI literacy, presents inherent limitations regarding the potential gap between perceived and actual capabilities. Participants may overestimate their competencies in areas such as prompt engineering or content evaluation, particularly when they lack objective benchmarks for assessment. Additionally, social desirability bias may be particularly pronounced when measuring ethical and compliance awareness, as respondents may provide socially acceptable responses rather than reflecting their actual behavioral tendencies in workplace contexts.

To address these self-report limitations, future research should explore multi-method validation approaches that combine subjective assessments with objective performance indicators. This could include developing standardized AI literacy tasks that measure actual prompt engineering effectiveness, content evaluation accuracy, and innovative application outcomes through behavioral observations rather than self-perceptions. Furthermore, incorporating peer evaluations, supervisor assessments, and objective performance metrics could provide convergent validity evidence and help distinguish between perceived and demonstrated AI literacy competencies.

Second, our cross-sectoral validation approach, while demonstrating broad applicability across public and private contexts during scale development, requires further validation across diverse organizational environments. Importantly, our empirical validation focused exclusively on Chinese public sector contexts, which may limit the generalizability of our theoretical relationships to private sector organizations where different performance pressures, innovation incentives, and regulatory environments may influence how GAIL translates into job performance. Future research should prioritize replicating our findings in private sector contexts, particularly in technology-intensive industries where AI adoption patterns and performance expectations may differ significantly from public sector environments.

Additionally, the specific requirements for GAIL may vary considerably across different industries and cultural contexts. For instance, in highly regulated industries such as healthcare or finance, ethical and compliance awareness may assume greater importance, while in technology development contexts, prompt engineering and innovative application capabilities may be more critical for performance outcomes. Future research should conduct industry-specific validation studies to identify sector-specific GAIL profiles and examine how different dimensional emphases contribute to performance in various professional contexts.

Third, this study employed a cross-sectional design with multi-wave data collection to analyze the mechanism by which GAIL influences job performance. While this approach helped reduce common method bias, it cannot establish definitive causal relationships between variables or capture the dynamic evolution of AI literacy over time. Generative AI Literacy may develop non-linearly as employees gain experience with AI technologies, and its effects on Creative Self-Efficacy and job performance may exhibit phase-specific characteristics that our cross-sectional approach cannot detect.

Future research should adopt longitudinal designs to track the long-term development of GAIL and its evolving impact on individuals and organizations across different stages of AI adoption. Such studies could reveal critical periods for literacy development, identify potential decay effects when AI technologies are not regularly used, and examine how the relationships between GAIL, Creative Self-Efficacy, and performance change as employees become more sophisticated AI users. Additionally, multi-level modeling approaches could explore the interactions between individual, team, and organizational factors in GAIL development, potentially identifying contextual moderators such as leadership support, organizational culture, and resource availability.

Fourth, while our research demonstrates the positive effects of GAIL on performance through enhanced Creative Self-Efficacy, we acknowledge that our study did not systematically explore potential negative consequences of AI literacy development. Increased proficiency with generative AI technologies may lead to over-reliance on AI systems, potentially diminishing critical thinking skills or creating unrealistic expectations about AI capabilities (Chaves-Maza & Fedriani, 2025). Furthermore, the rapid pace of AI technological evolution may create continuous learning pressure that generates stress and burnout among employees, particularly for those who struggle to keep pace with technological changes.

Future research should investigate the optimal balance between AI literacy development and maintaining human cognitive autonomy, examining when and how AI augmentation transitions from beneficial support to problematic dependency. Studies should also explore the psychological and organizational costs of maintaining high levels of GAIL in rapidly evolving technological environments, including the resource investments required for continuous training and the potential negative impacts on employee well-being.

Finally, future research should expand the cross-cultural validation of the GAIL framework to enhance its international applicability. While our findings suggest that the core theoretical relationships may have universal applicability, the specific manifestations of GAIL dimensions and their relative importance for performance may vary across cultural contexts with different values regarding technology adoption, hierarchy, and innovation. Comparative studies across diverse cultural and organizational contexts would help distinguish universal mechanisms from culture-specific applications, thereby enhancing the framework’s global relevance and enabling more effective cross-cultural AI literacy development programs.

Additionally, as generative AI technologies continue to evolve rapidly, future research should examine the dynamic adaptability of the GAIL framework to emerging AI capabilities and applications. This may include investigating how new AI functionalities influence the relative importance of different GAIL dimensions and whether additional competency areas should be integrated into the framework to maintain its relevance and predictive validity in evolving technological landscapes.

## Figures and Tables

**Table 1:** Comparative Review of GAIL Scale Development Studies.

Study	Core Dimensions	Target Population	Theoretical Foundation	Development Methods and Advantages	Key Differences from This Study	Identified Limitations
This Study	Basic technical proficiency, prompt optimization, quality evaluation, innovative practice, ethical and compliance awareness	Employees across workplace contexts	Digital literacy theory + workplace-specific requirements + expert validation	(1) AI-assisted item generation with expert validation; (2) cross-sectoral validation (private + public sectors); (3) workplace-specific competency focus; (4) process-oriented assessment approach	N/A—reference framework	(1) Specialized field applicability requires further testing; (2) long-term literacy evolution assessment needed
Nong et al. (2024)	Application ability, cognitive ability, morality, critical thinking, Self-Efficacy	General public	Technology acceptance model + multiple theories	(1) Large-scale surveys for representativeness; (2) interview-based need identification; (3) structural equation modeling validation	(1) Seven vs. five dimensions; (2) perceptual vs. behavioral focus; (3) general public vs. workplace context; (4) individual attitudes vs. collaborative performance	(1) Self-reported perceptions rather than actual competencies; (2) conceptual overlap between dimensions; (3) limited organizational context consideration
Ng et al. (2024)	Affective, Behavioral, Cognitive, and Ethical (ABCE)	Secondary school students	Educational psychology frameworks	(1) Combined interviews and pilot testing; (2) expert review for content validity; (3) 32-item validated questionnaire	(1) Educational vs. workplace context; (2) student focused vs. employee focused; (3) learning outcomes vs. performance competencies	(1) Limited technical operation focus; (2) cultural context specificity; (3) age-specific design limitations
Lee and Park (2024)	Technical proficiency, critical evaluation, communication proficiency, creative application, ethical competence	ChatGPT users	Digital literacy + user experience theory	(1) Delphi method for expert consensus; (2) focus group validation; (3) 25-item scale development	(1) ChatGPT-specific vs. general GAI; (2) user experience vs. workplace performance focus; (3) individual usage vs. organizational implementation	(1) Platform-specific limitations; (2) narrow generalizability; (3) limited workplace applicability
Zhao et al. (2022)	Knowing and Understanding AI, Applying AI, Evaluating AI Application, AI Ethics	Primary and secondary school teachers	Educational technology frameworks	(1) Teacher-specific needs assessment; (2) expert content validation; (3) structural equation modeling with 1013 participants	(1) Educational vs. workplace setting; (2) four vs. five dimensions; (3) teacher-specific vs. general employee focus	(1) Occupation-specific design; (2) limited cross-sector applicability; (3) educational context constraints

**Table 2:** Results of Exploratory Factor Analysis.

Dimension	AI-Generated Item	Factor Loading
Basic Operational Skills	I understand the principles and limitations of the AI I use.	0.911
	I can proficiently utilize the core and collaborative functions of AI tools.	0.896
	I can solve common technical problems encountered when using AI.	0.88
Prompt Engineering Ability	I can design effective AI prompts based on task requirements.	0.858
	I can optimize AI prompts by integrating technical terminology and examples.	0.843
	I can continuously refine AI prompts based on generated results and feedback.	0.869
Quality Evaluation Ability	I can assess the accuracy and reliability of AI-generated content.	0.896
	I can assess the consistency and coherence of AI-generated content.	0.881
	I can evaluate the logic and completeness of AI-generated content.	0.883
Innovative Application Ability	I can identify and capitalize on innovative opportunities in my work through AI.	0.874
	I can generate creative and innovative ideas for my work using AI.	0.827
	I can transform innovative ideas into tangible results using AI.	0.832
Compliance and Ethical Awareness	I can avoid ethical risks when using AI.	0.895
	I can ensure the protection of privacy and sensitive data when using AI.	0.869
	I can comply with laws and regulations related to AI usage.	0.894
	I can follow organizational guidelines when using AI.	0.89
	I can adhere to professional ethics when using AI.	0.859

Note: To reduce the reading burden on respondents and simplify the scale, the term “Generative Artificial Intelligence” is consistently abbreviated as “AI” throughout the questionnaire, with a clear explanation provided prior to the respondents beginning the survey. Additionally, before the formal answering process begins, the questionnaire offers a basic introduction to Generative AI to help respondents understand the relevant concepts. A skip option is also included: if respondents indicate that they have never used Generative AI, the questionnaire will end immediately.

**Table 3:** AVE and CR Values For the Five Dimensions of Generative AI Literacy in the Workplace.

Construct Dimension	AVE	CR
Basic Operational Skills	0.825	0.934
Prompt Engineering Ability	0.786	0.917
Quality Evaluation Ability	0.808	0.927
Innovative Application Ability	0.776	0.912
Ethical and Regulatory Awareness	0.799	0.952

**Table 4:** Reliability and Validity Testing.

Variable	CR	AVE	Cronbach’s α
Generative AI Literacy	0.868	0.564	0.943
Creative Self-Efficacy	0.917	0.579	0.916
Job Performance	0.885	0.658	0.885

**Table 5:** Confirmatory Factor Analysis.

Model	*χ* ^2^	*df*	CFI	TLI	SRMR	RMSEA
Three-Factor Model (GAIL, CSE, JP)	402.963	369	0.996	0.995	0.033	0.016
Two-Factor Model (GAIL + CSE, JP)	621.869	371	0.968	0.965	0.074	0.044
One-Factor Model (GAIL + CSE + JP)	739.038	372	0.954	0.950	0.071	0.054

**Table 6:** Descriptive Statistics and Correlation Analysis Between Variables.

Variable	1	2	3	4	5	6	7	8	9	10	11	12	13
Mean	4.026	0.52	3.006	2.433	2.422	3.047	3.09	2.503	2.439	2.462	4.174	4.021	4.58
Standard Deviation	0.813	0.5	1.429	1.143	1.143	1.374	1.398	1.05	1.102	1.122	0.995	0.996	1.244
1. Usage Frequency	1												
2. Gender	0.110 *	1											
3. Age	−0.010	−0.021	1										
4. Marital Status	0.050	0.007	−0.016	1									
5. Education Level	0.073	0.028	0.002	0.025	1								
6. Work Experience	0.156 **	0.024	0.024	0.006	0.121 *	1							
7. Political Affiliation	−0.066	0.104	−0.028	0.012	−0.020	−0.031	1						
8. Monthly Income	0.391 ***	0.133 *	−0.045	0.010	0.190 ***	0.170 **	−0.071	1					
9. Job Type	−0.013	0.013	−0.015	−0.003	0.003	−0.031	0.029	−0.116 *	1				
10. Job Rank	0.159 **	−0.030	0.004	0.000	0.050	0.122 *	−0.014	0.206 ***	−0.056	1			
11. Generative AI Literacy	0.540 ***	0.081	0.075	0.007	0.038	0.168 **	−0.044	0.421 ***	−0.047	0.150 **	1		
12. Creative Self-Efficacy	0.539 ***	0.067	0.053	0.024	0.052	0.156 **	−0.050	0.404 ***	−0.059	0.119 *	0.661 ***	1	
13. Job Performance	0.633 ***	0.125 *	0.030	0.081	0.135 *	0.252 ***	−0.039	0.493 ***	−0.041	0.275 ***	0.706 ***	0.742 ***	1

Note: * *p* < 0.05, ** *p* < 0.01, *** *p* < 0.001.

**Table 7:** Hierarchical Regression Analysis of Direct and Mediated Effects.

Variable	Creative Self-Efficacy		Job Performance			
	Model 1	Model 2	Model 3	Model 4	Model 5	Model 6
1. Usage Frequency	0.572	0.126	0.744 ***	0.273 **	0.337 ***	0.207 *
2. Gender	−0.026	−0.002	0.09	0.117	0.109	0.118
3. Age	0.049	0.018	0.04	0.006	0.005	−0.003
4. Marital Status	0.000	0.013	0.054	0.068	0.055	0.061
5. Education Level	−0.026	−0.009	0.035	0.052	0.052	0.057
6. Work Experience	0.039	0.022	0.1 **	0.084 *	0.075 *	0.072 *
7. Political Affiliation	0.001	−0.005	0.011	0.005	0.010	0.008
8. Monthly Income	0.226 ***	0.141 **	0.292 ***	0.203 ***	0.144 **	0.129 *
9. Job Type	−0.024	−0.013	0.005	0.016	0.021	0.023
10. Job Rank	−0.004	−0.005	0.146 ***	0.145 ***	0.148 ***	0.148 ***
11. Generative AI Literacy		0.640 ***		0.680 ***		0.554 ***
12. Creative Self-Efficacy					0.713 ***	0.320 **
R^2^	0.374 ***	0.462 ***	0.573 ***	0.619 ***	0.741 ***	0.766 ***
F	9.12	6.901	15.473	8.561	14.361	7.08

Note: * *p* < 0.05, ** *p* < 0.01, *** *p* < 0.001.

**Table 8:** Mediating Effect Test Based on the Bootstrap Method.

Effect Path	Effect Value	Standard Error	Lower Bound	Upper Bound
Direct Effect				
GAIL → JP	0.365	0.073	0.245	0.532
Indirect Effect				
GAIL → CSE → JP	0.537	0.132	0.348	0.838
Total Effect	0.680	0.099	0.495	0.895

## Data Availability

The datasets analyzed during the current study are available from the corresponding author on reasonable request.
